# Anti-allergic and anti-inflammatory effects of *Gastrodia elata* Blume extract in ovalbumin-induced asthma rat model

**DOI:** 10.1186/s42826-025-00252-8

**Published:** 2025-09-16

**Authors:** Jeong Su Park, Yeon Su Lee, Da Eun Jung, Ji Won Seo, Hyeon Jeong Na, Jin Woo Hong, Jae-Ho Shin

**Affiliations:** Department of Biomedical Laboratory Science, Graduate School, Eulji University, Seongnam, 13156 Korea

**Keywords:** *Gastrodia elata* Blume, Allergic asthma, Anti-inflammation, Anti-allergic, Ovalbumin, Rat

## Abstract

**Background:**

Allergic asthma is a chronic inflammatory disease in which bronchial inflammation causes narrowing of the bronchi when exposed to allergens, resulting in coughing, wheezing, and difficulty breathing. *Gastrodia elata* Blume (GEB) is a perennial Orchidaceae plant native to alpine areas and is known to be effective in anti-inflammatory and anticonvulsants. This study evaluated the anti-inflammatory and anti-allergic effects of GEB extract in a rat model of allergic asthma induced by ovalbumin. This study evaluated the anti-inflammatory and anti-allergic effects of GEB extract in a rat model of allergic asthma induced by ovalbumin. Twenty-four 6-week-old Wistar rats were divided into three groups: control group (CON), ovalbumin (OVA) -induced group, and GEB treatment group. Except for the CON group, the remaining groups were sensitized to OVA by intraperitoneal injection, and asthma was induced by OVA intranasal instillation. The CON and OVA groups were administered distilled water, and the GEB group was administered 7 g/kg of GEB extract for 11 days.

**Results:**

Serum total IgE levels were decreased in the GEB group compared to the OVA group. Also, lung IL-4, IL-5, and IL-13 levels were significantly lower in the GEB group than in the OVA group. Histopathological analysis using hematoxylin and eosin and periodic acid Schiff staining, the tracheal and alveolar walls of the OVA group were thickened, and there was increased infiltration of inflammatory cells in the bronchi, perivascular, and alveolar spaces. As for lung damage caused by OVA, GEB treatment reduced the infiltration of inflammatory cells into the bronchi and blood vessels, and the alveolar spaces were maintained, showing a structure similar to that of the CON group. Immunohistochemical analysis showed that IL-4, IL-5, CD206, and MPO expression levels were reduced in the GEB group compared to the OVA group.

**Conclusions:**

This suggests that GEB treatment has an anti-inflammatory and anti-allergic effect by reducing the levels of IgE and the cytokines IL-4, IL-5, and IL-13 and ameliorating histopathological changes in an asthma rat model.

**Supplementary Information:**

The online version contains supplementary material available at 10.1186/s42826-025-00252-8.

## Background

Asthma affects approximately 300 million people worldwide, leading to numerous deaths each year. Despite advances in medical technology, the global impact of asthma remains high. Allergic asthma is a chronic inflammatory disease of the airways, typically triggered by inhalation of allergens and characterized by increased mast cells, eosinophils, macrophages, and neutrophils [1–3]. This disease causes recurrent symptoms of wheezing, breathlessness, chest tightness, and coughing at night or daybreak, in severe cases, lung function may decline, and breathing difficulties may even progress to respiratory failure [1, 4]. It occurs particularly frequently in childhood and is characterized by airway obstruction, airway hyperresponsiveness, and airway inflammation resulting from exposure to various irritants [5]. Type 1 allergy causes asthma through an immune response to allergens mediated by T helper 2 (Th2) cells [6–8]. To mimic the pathogenesis of human asthma and evaluate the safety and efficacy of treatment, asthma was induced in animal models such as mice, rats, and guinea pigs using various allergens such as ovalbumin (OVA), house dust mites, and molds [9, 10]. Among them, OVA is the primary protein found in chicken egg whites. It is known to be the most commonly used allergen in asthma experiments because it causes severe allergic lung inflammation with features similar to human asthma [10, 11]. When an allergen enters the body, it is processed by macrophages, natural killer (NK) cells, and dendritic cells and produces cytokines such as interleukin-4 (IL-4), IL-5, and IL-13 [12, 13]. The cytokines activate B cells to secrete immunoglobulins, induce class switching from IgM to IgE, and promote differentiation into M2 macrophages. Cytokines and IgE secreted in this way cause inflammation and lung damage [14–16].

Inhaled corticosteroids are used to treat allergic asthma, a chronic inflammatory disease, but they can cause side effects such as dysphonia and candidiasis [17, 18]. For this reason, it is necessary to find natural substances that can help prevent and improve asthma and have fewer side effects. Many natural products, including Kanakasaba[19], Anatabine [20], and *Olea europaea* L [21], have been studied for the treatment and prevention of respiratory diseases such as asthma. *Gastrodia elata* Blume (GEB) is a plant from the Orchidaceae plant and is distributed in mountainous regions of East Asia, including Korea, China, and Japan. It is currently used clinically to treat dizziness, headaches, epilepsy, convulsions, and high blood pressure [22, 23]. Additionally, recent studies have shown that GEB extract prepared with 70% ethanol has anti-inflammatory effects [24]. Phenolic compounds found in GEB extract, such as 4-hydroxybenzaldehyde, 4-hydroxy benzyl alcohol, benzyl alcohol, bis-(4-hydroxyphenyl)methane, 4-(4′-hydroxybenzyloxy)benzyl methyl ether, 4-hydroxy-3-methoxybenzyl alcohol, 4-hydroxy-3-methoxybenzaldehyde, 4-hydroxy-3-methoxybenzoic acid, and 1,2-bis (4-(β-D-glucopyranosyloxy)benzyl) citrate (parishin), exhibit antiasthmatic effects in a guinea pig asthma model [25]. Like this, the main active ingredients of GEB have been reported to have various pharmacological activities such as neuroprotection, anti-inflammatory, and anti-asthma. However, the anti-inflammatory and anti-allergic actions of GEB extract in asthma rat models have not been identified.

To date, the anti-inflammatory and anti-allergic effects of GEB extract in asthma models have not been clearly elucidated. Given the growing interest in natural product-based health functional foods with fewer side effects, it is necessary to clarify the pharmacological effects of GEB on allergic asthma for its potential use as a functional food ingredient. Therefore, this study investigated the histopathological changes, immune cells, and cytokines in lung tissues to evaluate the potential protective effects of GEB in an OVA-induced allergic asthma model that is very similar to the pathophysiology of human asthma. Through this approach, we aimed to elucidate the potential of GEB as a novel natural anti-asthmatic agent and contribute to its utilization as a safer and more effective health functional food for allergic diseases.

## Methods

### Preparation of GEB extract

GEB was provided by the Rural Development Administration (Jeonju, Jeonbuk State, Korea). The GEB was dried using hot air at 70 °C. The dried GEB was coarse-ground. Distilled water (DW) 10 times the weight (g) of coarse-ground GEB was added, extracted four times at 96 °C, and the first to fourth extracts were mixed. DW was added to the extract, and the mixture was concentrated under reduced pressure and then lyophilized. The lyophilized GEB extract was sterilized through irradiation. To analyze the components of GEB, 1 mL of GEB extract and 4 mL of 50% methanol were added to the vortex for 10 s. The mixed samples were subjected to ultrasonic extraction for 30 min. The ultrasonically extracted sample was centrifuged at 10,000 rpm for 10 min, and the supernatant was separated and filtered through a 0.45 μm syringe filter. Afterward, the GEB extract was analyzed using HPLC–DAD (High-performance liquid chromatography-diode array detector) (Table 1). GEB extract was freshly prepared by completely dissolving 7 g of GEB powder in 10 mL of DW at the time of the experiment.

**Table 1:** HPLC–DAD instrumental analysis conditions for the analysis of GEB extract

LC (Liquid chromatography)		Agilent 1100 series						
Column		Capcellpak C18 UG1200 (4.6 × 250 nm, 5 µm)						
Mobile phase		A: Water: ACN = 99:1 B: ACN						
Gradient mode		Time (min)	0	15	20	25	25.1	30
		B (%)	0	0	90	90	90	0
Flow		1.2 mL/min						
Injection volume		10 µL						
Detector	DAD detector	270 nm						

### Chemicals and reagents

Albumin from chicken egg white (OVA, A5378-1G) and aluminum hydroxide (Alum, 239,186-25G) were purchased from Sigma-Aldrich (St Louis, MO, USA). Rat interleukin-4 (IL-4, CSB-E04635r), interleukin-5 (IL-5, CSB-E07435r) and interleukin-13 (IL-13, CSB-E07454r), enzyme-linked immunosorbent assay (ELISA) kits were obtained from Cusabio (Wuhan, P.R. China), and rat total IgE ELISA kit (ab157736) was obtained from Abcam (Inc., Cambridge, MA, USA). To perform immunohistochemistry (IHC), anti-IL-4 antibody (ab9811, Abcam), anti-IL-5 antibody (LS-B14355, LSbio, Seattle, WA, USA), anti-CD206 antibody (ab64693, Abcam), anti-myeloperoxidase antibody (ab208670, Abcam), and ABC-horseradish peroxidase (HRP) kit (PK6101) was obtained from Vector Laboratories (Burlingame, CA, USA) and 3,3'-diaminobenzidine (DAB) Substrate Kit (34,002) was purchased from Thermo Fisher Scientific (Waltham, MA, USA).

### Animals

Male Wistar rats (6 weeks old, 180–230 g) were purchased from Orient Bio (Seongnam, Gyeonggi-do, Korea). The animals were housed in cages measuring 260 mm (W) × 420 mm (D) × 180 mm (H) (2–3 rats per cage) with free access to food (24.52% protein, 12.41% fat, 63.07% carbohydrates) and tap water and were maintained under the following conditions: temperature, 22 ± 2℃; Humidity, 50± 10%; circadian cycle, 12-h light/dark cycle. The body weight (BW) and food and water intake were measured twice a week during the experimental period.

### Experimental design

After 1 week of adaptation to standard housing conditions, animals were randomly divided into 3 groups with 8 rats per group: ⅰ) Control (CON) group, ⅱ) OVA-induced asthma group, and ⅲ) GEB-treated group (Supplementary Fig. 1).

On days 1, 2, 3, and 11, the OVA and GEB groups were sensitized by dissolving 200 μg of OVA in 1 mL of saline containing 10 mg of alum as an adjuvant, and the CON group received the same volume of saline with 10 mg of alum added. On days 20, 21, and 22, 100 μL of 1% OVA in saline was intranasally instilled in the OVA and GEB groups, while the CON group received the same amount of saline intranasally. GEB extract was administered orally once daily from day 11 to day 23 at the maximum nontoxic dose of 7 g/kg. Twenty-four hours after the last administration, the rats were fasted overnight and sacrificed under anesthesia using isoflurane (Halocarbon, Georgia, USA). Blood was collected from the abdominal aorta. Blood samples were placed in a serum-separating tube (SST) and centrifuged at 3,000 rpm and 4 °C for 15 min to obtain the serum. The serum was stored at −80 °C for biochemical analysis. The left lung was stored in 10% neutral buffered formalin solution for histopathological and immunostaining analysis, and the right lung was stored at −80 °C for biochemical analysis.

### Bronchoalveolar lavage fluid (BALF) collection and differential cell counts

To collect BALF, rats were slowly instilled with 3 mL of cold phosphate-buffered saline (PBS) three times after endotracheal intubation. This resulted in a collected average volume of 1.95 ± 0.04 mL of BALF. The collected BALF was centrifuged at 2,000 rpm and 4 °C for 5 min. After centrifugation, all supernatants were collected and stored at −80 °C, and the cell pellets were suspended in 100 μL of PBS. Subsequently, 3 × 10^4^ cells were added to the slide in 200 μL of PBS and centrifuged using cytospin (Cellspine, Hanil, Gimpo, Korea) equipment. After drying, the slides were stained with Diff-Quik reagent (38,721, Sysmex, Kobe, Japan). To observe changes in the number of eosinophils and neutrophils in BALF, the total number of eosinophils and neutrophils was counted in four fields of view on Diff-Quik stained slides.

### Measurement of total IgE in serum

Total IgE levels were measured according to the ELISA manufacturer's instructions using serum stored at −80 °C. Absorbance was measured at 450 nm using SkanIt™ software for a microplate reader (Thermo Fisher Scientific).

### Measurement of cytokines in lung

To measure the cytokines IL-4, IL-5, and IL-13 in lung homogenates, in ice, 100 mg of lung tissue was homogenized in 1 mL of PBS and stored overnight at −20 °C. After subjecting the homogenate to two freeze–thaw cycles to break the cell membrane, it was centrifuged at 5,000 xg and 4 °C for 5 min. The obtained supernatant was used and measured according to the ELISA manufacturer's instructions. Absorbance was measured at 450 nm using SkanIt™ software for a microplate reader (Thermo Fisher Scientific).

### Histopathological analysis

The fixed lung tissue was dehydrated with alcohol, washed with xylene, and embedded in paraffin. The blocks were then cut into 4 μm thick sections. The sections were stained with hematoxylin and eosin (H&E) and periodic acid-Schiff (PAS), mounted with Permount, and observed using a light microscope (Olympus, Tokyo, Japan). Lungs were observed on H&E-stained slides to evaluate peribronchial inflammatory cell infiltration, alveolar inflammatory cell infiltration, and alveolar wall changes. The lung inflammation score was performed in a blinded manner following the method described by Zeng Z et al. [26]. The degree of lung inflammation was evaluated using the following scoring system: 0 = no bronchial and perivascular inflammatory cells; 1 = few scattered bronchial and perivascular inflammatory cells; 2 = focal inflammatory cells around the bronchi and blood vessels; 3 = one layer of inflammatory cells encircling the bronchi and blood vessels; 4 = two or more layers of inflammatory cells encircling the bronchi and blood vessels (Table 2). PAS staining, which stains neutral mucus, was performed to confirm mucus secretion caused by inflammation in the bronchi.

**Table 2:** Scoring methods for histopathological observation

Grade	Diagnostic criteria
0	No bronchial and perivascular inflammatory cells
1	Few scattered bronchial and perivascular inflammatory cells
2	Focal inflammatory cells around the bronchi and blood vessels
3	One layer of inflammatory cells encircling the bronchi and blood vessels
4	Two or more layers of inflammatory cells encircling the bronchi and blood vessels

### Immunohistochemical analysis

IL-4, IL-5, CD206, and MPO expression levels associated with inflammatory cells, such as eosinophils, neutrophils, and macrophages, were evaluated in lung tissues using immunohistochemistry. The lung tissue was sectioned at 4 μm thickness on silane-coated slides. After deparaffinization and dehydration, the sections were boiled in a microwave for 10 min using citrate buffer (pH 6.0) for antigen retrieval and then cooled at room temperature for 30 min. Anti-IL-4 antibody (1:500), anti-IL-5 antibody (1:1000), anti-myeloperoxidase antibody (1:1000), CD206 antibody (1:1000), and MPO antibody (1:1000) were incubated overnight at 4 °C as primary antibody. The samples were visualized using 3,3'-diaminobenzidine tetrahydrochloride (DAB), counterstained with Mayer's hematoxylin, and examined using a light microscope.

### Statistical analysis

The significance of the differences in mean values among the experimental groups was determined using a one-way analysis of variance (ANOVA), followed by Tukey's HSD test. The level of statistical significance was set at *p* < 0.05. SPSS (version 28.0; SPSS Inc., Chicago, USA) calculated probability values. All the results in this study are expressed as mean ± standard deviation (SD).

## Results

### Active ingredient content of GEB extract using HPLC–DAD

**Fig. 1:**
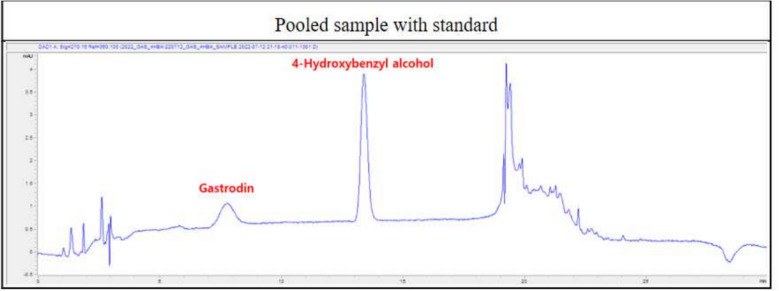
Active ingredients of GEB extract

GEB extract contains the active ingredients known as 4-hydroxybenzyl alcohol and gastrodin (Fig. 1).

### Effects of GEB on inflammatory cell counts in BALF

**Fig. 2:**
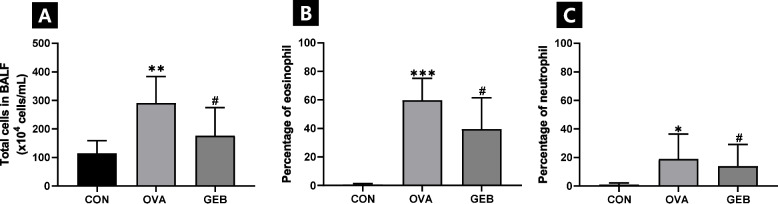
Effect of GEB on inflammatory cells in asthmatic rats. Mean values ​​of (**A**) total cell counts in BALF, (**B**) percentage of eosinophil, and (**C**) percentage of neutrophil. Values are expressed as mean ± SD of 8 rats per group. Compared with the CON group, *p < 0.05, **p < 0.01, ***p < 0.001; compared with the OVA group, ^#^*p* < 0.05

The total cell count in BALF was significantly increased in the OVA group compared to the CON group and significantly decreased in the GEB group compared to the OVA group (Fig. 2A). Additionally, when checking the asthma status of rats after drug treatment for asthma caused by OVA administration in BALF, eosinophils, and neutrophils, which are generally increased in asthma diseases, were scarcely found in the CON group. Eosinophils and neutrophils were found in the OVA and GEB groups administered OVA, but the levels of eosinophils and neutrophils were markedly reduced in the GEB group compared to the OVA group (Fig. 2B, C).

### Effects of GEB on the total IgE in serum

**Fig. 3:**
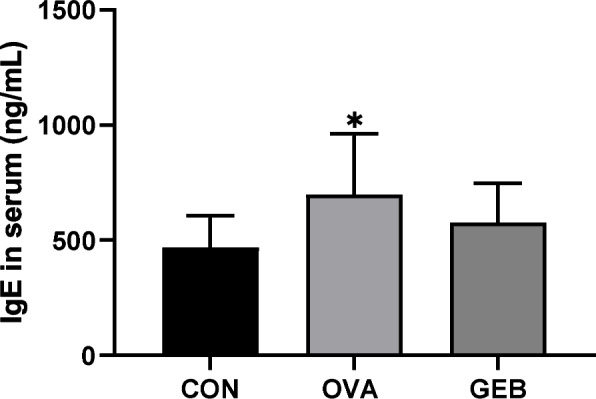
Total IgE level in serum. Total IgE levels in serum were quantified using enzyme-linked immunosorbent assay (ELISA). Values were mean ± SD of 8 rats per group. Compared with the CON group, *p < 0.05

The total IgE level in the serum of the OVA group was significantly higher than that of the CON group. The total IgE level in the GEB group tended to decrease compared to the OVA group. GEB treatment has been shown to reduce IgE production due to the inhibition of allergic responses (Fig. 3).

### Effects of GEB on cytokines in the lung

**Fig. 4:**
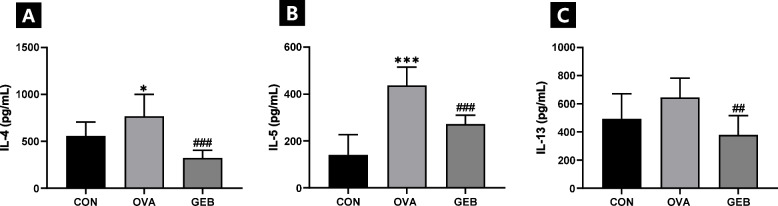
Changes in cytokines in the lung. Levels of cytokines (**A**) IL-4, (**B**) IL-5, and (**C**) IL-13 were measured using ELISA in lung homogenates of OVA-induced asthmatic rats. Values were mean ± SD of 8 rats per group. Compared with the CON group, *p < 0.05, ***p < 0.001; compared with the OVA group, ^##^*p* < 0.01, ^###^*p* < 0.001

To analyze type 2 cytokines induced by GEB in rats with induced asthma, we examined type 2 cytokine levels in the lungs of rats administered GEB. In the OVA group, the levels of IL-4 (Fig. 4A), IL-5 (Fig. 4B), and IL-13 (Fig. 4C) were dramatically increased in contrast to the CON group. Compared with the OVA group, the levels of IL-4, IL-5, and IL-13 in the GEB group were significantly downregulated.

### Effects of GEB on airway inflammation and mucus secretion in lung

**Fig. 5:**
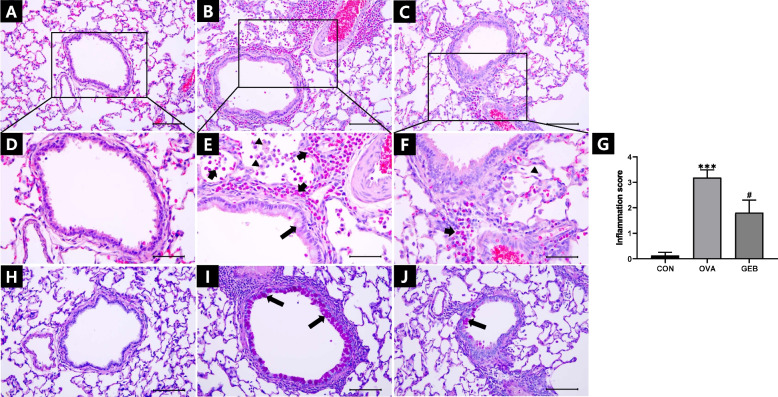
Histopathological changes in H&E and PAS stained lung. Representative tissue sections of the lung were stained with H&E and PAS. (**A**-**F**) H&E, (**H**-**J**) PAS, (**A**, **D**, **H**) CON group, (**B**, **E**, **I**) OVA group, (**C**, **F**, **J**) GEB group, (**G**) Inflammatory cell scoring results. The infiltration of inflammatory cells such as eosinophils in the bronchial, perivascular, and alveolar spaces (arrowheads), goblet cells (long arrows), and alveolar macrophages (short arrows). PAS-positive cells (long arrows). Scale bars in A-C and H-J are 100 μm, and in D-F, 50 μm, each. Values were mean ± SD of 8 rats per group. Compared with the CON group, ***p < 0.001; compared with the OVA group, ^#^*p* < 0.05

H&E staining showed normal structures of the bronchi and lungs in the CON group (Fig. 5A, D). The tracheal and alveolar walls of the OVA group were thickened, and there was increased infiltration of inflammatory cells in the bronchi, perivascular, and alveolar spaces (Fig. 5B, E). OVA-induced damage was ameliorated by GEB treatment. The GEB treatment reduced inflammatory cell infiltration into blood vessels, bronchioles, and alveolar spaces, and alveolar spaces were maintained. GEB treatment reduced inflammatory cells and structural changes in the alveolar space of the lung and tracheal tissue (Fig. 5C, F). The lung inflammation score was markedly higher in the OVA group compared with the CON group. However, the OVA group had dramatically lower scores than the GEB group (Fig. 5G). As a result of confirming histopathological changes in lung tissue stained with PAS, normal mucus production was not observed in the bronchioles of the CON group, but an increase in mucus production was observed in the bronchioles of the OVA group (Fig. 5H, I). In contrast, bronchioles in the GEB group showed decreased mucus production as inflammation decreased (Fig. 5J).

### Effects of GEB on the expression of cytokines, CD206, and MPO in the lung

**Fig. 6:**
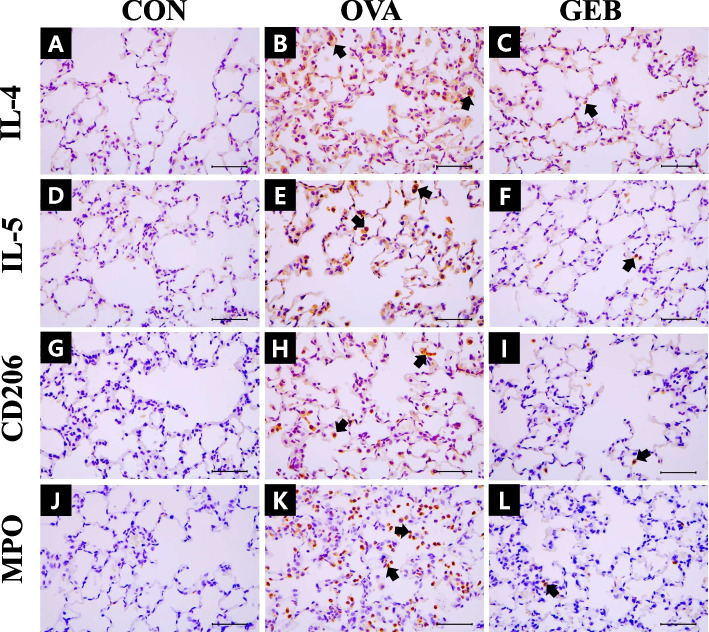
IL-4, IL-5, CD206, and MPO expression in immunohistochemical staining of the lung. A Representative histopathological section of the lungs was stained by immunohistochemistry. (**A**-**C**) IL-4, (**D**-**F**) IL-5, (**G**-**I**) CD206, (**J**-**L**) MPO, (**A**, **D**, **G**, **J**) CON group, (**B**, **E**, **H**, **K**) OVA group, (**C**, **F**, **I**, **L**) GEB group. IL-4, IL-5, CD206, and MPO-positive cells (arrows). Scale bars in 50 μm

Immunohistochemical staining was performed to confirm the expression of cytokines and the M2 macrophage marker CD206 in the lung tissue of OVA-induced asthmatic rats. The expression of IL-4 in the OVA group was higher than in the CON group (Fig. 6B). Compared with the OVA group, the expression in the GEB group was lower (Fig. 6C). IL-5, a key mediator of eosinophil activation, was upregulated in the OVA group compared to the CON group (Fig. 6E). The expression of IL-5 was lower in the GEB group (Fig. 6F). The expression of CD206, an M2 macrophage marker, was increased in the OVA group (Fig. 6H). On the other hand, the expression of CD206 was downregulated in the GEB group (Fig. 6I). The expression of MPO, an enzyme contributing to neutrophil formation, was increased in the OVA group compared to the CON group. This increase was reduced in the GEB group (Fig. 6K, L).

## Discussion

Allergic asthma is a chronic respiratory disease characterized by the contraction of bronchial smooth muscles and an increase in inflammatory cells and cytokines due to chronic inflammation triggered by external allergen substances [27, 28]. Inflammation occurs in the body during tissue damage processes such as cell death, cancer, ischemia, and degeneration [29, 30]. It is known that when asthma is induced in rats, inflammatory cells, cytokines, and lung damage increase due to inflammation [7, 31–33]. This study aimed to determine if GEB treatment could reduce inflammatory cells and cytokines, as well as alleviate lung damage in OVA-induced rats.

Eosinophils are known to increase in parasitic infections, autoimmune diseases such as rheumatoid arthritis and systemic lupus erythematosus, and allergic diseases such as asthma, while neutrophils are known to increase due to allergies or immune reactions [34, 35]. In this experiment, we found dramatic increases in total cell counts, eosinophil counts, and neutrophil counts in the BALF of OVA-induced asthmatic rats. However, the inflammatory response to OVA was mitigated with GEB treatment. In addition, the cytological findings of BALF are consistent with the results of Chen YR et al. [36], who reported an increase in the total cell count, eosinophil count, and neutrophil count in the BALF of mice administered OVA and decreased after treatment. Activation of eosinophils triggers the release of histamine and type 2 cytokines from mast cells, and the released histamine can activate other immune cells, such as neutrophils and alveolar macrophages, promoting inflammation in asthma [37]. It is known that when asthma is induced by OVA, smooth muscle hypertrophy occurs in lung tissue, along with inflammatory cell infiltration around the bronchi, blood vessels, and alveoli [38–40]. In the lung structure of OVA-induced asthmatic rats, numerous inflammatory cells were observed in the bronchi, blood vessels, and alveolar spaces, and inflammatory cells infiltrated around the alveoli, narrowing the alveolar space and increasing alveolar macrophages. GEB treatment maintained alveolar space by reducing inflammatory cells in the bronchi, blood vessels, and surrounding alveoli, as well as reduced alveolar macrophages. This was consistent with the results of BALF inflammatory cells. In the allergic asthma pathway, airway hyperresponsiveness is closely associated with elevated eosinophil levels in the lungs. The reduction of inflammatory cells in BALF and lung tissue indicates that they play an important role in the treatment of allergen-induced bronchial hyperresponsiveness [19].

A representative disease demonstrating a Th2 dominant response is allergic asthma. When naive T cells are stimulated by a specific allergen, they differentiate into Th2 cells and produce type 2 cytokines IL-4, IL-5, and IL-13, which promote airway eosinophilia, excessive mucus production, bronchial hyperresponsiveness, and IgE synthesis [41, 42].

The generated IgE binds to the high-affinity IgE receptor on the surface of mast cells and degranulates them, and the degranulated cells release histamine and cytokines to induce mucus production and bronchial remodeling [12]. We determined whether treatment with GEB reduces total IgE levels in the serum of OVA-induced asthma rats. Total IgE levels in the serum of OVA-induced asthmatic rats increased, while GEB treatment decreased total IgE levels. A cytokine closely related to IgE is IL-4, a key cytokine in allergic diseases that mainly acts in the early stages of asthma and promotes the process of synthesizing IgE and replacing macrophages with M2 cells [43, 44]. In addition, studies have reported that blocking the IL-4 pathway can reduce serum total IgE levels in rats [45, 46]. IL-4 levels in lung from OVA-induced asthma rats were significantly increased compared to controls and markedly decreased by GEB treatment. This indicates that GEB suppresses the activation of B cells into plasma cells by reducing the level of IL-4, a type 2 cytokine, which was stimulated by OVA, thereby reducing IgE production and alleviating allergic reactions.

IL-5 is produced by Th2 cells, mast cells, airway smooth muscle, and epithelial cells, and it is associated with increased eosinophils during allergies or parasitic infections [47, 48]. The link between IL-5 and eosinophilic inflammation has been demonstrated in animal and human asthma experiments, where inhibition of IL-5 with monoclonal antibodies reduced eosinophils caused by allergens or chronic diseases [47]. Additionally, when OVA was induced, an IL-5-dependent eosinophil influx was observed in BALF and lung tissue [49]. This study found that treating OVA-infected rats with GEB could significantly reduce eosinophil counts in BALF and lungs by suppressing IL-5 levels. Consistent with our report, Ou G et al. [50] found that the levels of IL-5 and eosinophils were significantly reduced in OVA mice.

IL-13 is primarily involved in the later stages of allergic reactions and is a cytokine secreted by CD4 cells, NK T cells, mast cells, and eosinophils. IL-13 induces tissue remodeling, regulates barrier function, induces differentiation and proliferation of goblet cells, and promotes mucus production [51]. Goblet cell hyperplasia and excessive mucus production in the airway epithelium are characteristic of asthma and obstructive pulmonary disease, primarily due to an increase in eosinophils, which cause tissue damage and inflammation, leading the lungs to secrete mucus for protection [52, 53]. The association of IL-13 with goblet cell production and mucus secretion has already been demonstrated in animal models to influence monocyte infiltration, goblet cell proliferation, acid and neutral mucus overproduction, epithelial airway fibrosis, and crystal precipitation [54]. In chronic asthma, persistent airway inflammation caused by OVA leads to airway remodeling. Our results showed that GEB could significantly reduce the level of IL-13 in the lung. Bronchial epithelial cells serve as an important barrier against invading allergens. These cells secrete mucus, which becomes stuck to dust and is then expelled through the ciliary movement of the respiratory tract [55]. However, allergens can stimulate an inflammatory response in bronchial epithelial cells, causing them to differentiate into goblet cells, which release large amounts of mucus that block the airways, causing breathing difficulties and even death from suffocation [56]. We confirmed through PAS staining that GEB treatment reduced mucus secretion in OVA-induced asthma rats. Our results showed that GEB could significantly reduce the levels of IL-13 and mucus secretion. This shows that GEB treatment has the potential to improve airway inflammation by regulating airway remodeling through inhibition of the IL-13 pathway.

Alveolar macrophages are divided into M1 cells and M2 cells based on the stimulator. M1 macrophages are predominantly activated in models of acute lung injury and are stimulated by interferon-γ (IFN-γ) and Lipopolysaccharide (LPS) to induce non-allergic immune responses [16, 57]. Meanwhile, the macrophages that are primarily activated in allergic asthma models are M2 macrophages [9]. Alveolar macrophages differentiate into M2 macrophages by IL-4 and IL-13, which induce allergic immune responses and are involved in apoptosis and removal of necrotic tissue [58]. CD206 is a mannose receptor expressed in M2 macrophages and serves as a marker of M2 macrophages [59]. Neutrophils, the first immune cells to respond when inflammation occurs, are a cell type found during inflammation and airway narrowing in asthma. When they accumulate in the lung inflammation site, monocytes from the blood are recruited and differentiate into macrophages[35, 60, 61]. MPO is a heme-containing peroxidase expressed primarily in neutrophils and less frequently in monocytes[62]. In asthma patients, IL-4 and IL-5 are secreted, which generate eosinophils and induce airway inflammation and airway remodeling [63, 64]. Our experiments showed that GEB treatment suppressed the expression of IL-4 and IL-5 and decreased the expression of CD206, a marker of M2 macrophages, and MPO, which is involved in neutrophil production. Therefore, we have confirmed that GEB can improve lung function by protecting against inflammation in asthmatic rats.

**Fig. 7:**
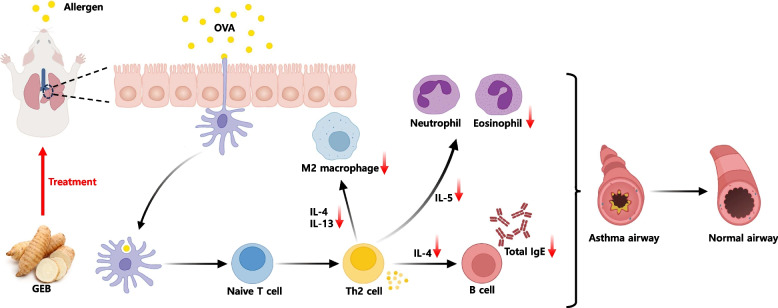
The suggested mechanisms on the anti-allergic and anti-inflammatory effects of GEB extract

Asthma is a lung disease characterized by lung inflammation due to persistent stimulation by allergens. When allergens enter, naive T cells are stimulated to differentiate into Th2 cells and secrete type 2 cytokines. Type 2 cytokines stimulate the production of IgE, an important immunoglobulin in allergic diseases, and M2 macrophages and are involved in airway remodeling, mucus secretion, and inflammatory cell infiltration into the airways. In Our data, GEB reduced the levels of type 2 cytokines, decreased inflammatory cells in the airway and lung, reduced mucus secretion, and suppressed the production of IgE and M2 macrophages (Fig. 7). The main active ingredients in GEB extract are 4-hydroxybenzyl alcohol and gastrodin. Among them, 4-hydroxybenzyl alcohol has been proven to have an anti-allergic effect in a guinea pig asthma model [25], and gastrodin has been proven to have an anti-inflammatory effect in several studies [65–67]. In our study, the GEB extract containing these active ingredients demonstrated anti-allergic and anti-inflammatory effects in asthma. Therefore, GEB extract has been proven to be a natural product that can relieve allergic asthma. However, the current concentration of GEB extract is the maximum dose that can be administered without toxicity, but it is too high to be safely applied to humans. Future research is needed to reduce the total amount of this extract and make it more concentrated so that it can be applied to humans.

## Conclusions

We demonstrated that treatment of OVA-induced rats with GEB has the potential to alleviate asthma symptoms, such as airway inflammation and mucus hypersecretion, by reducing type 2 cytokines, IgE, and inflammatory cells. In conclusion, this study shows that GEB is a promising candidate for improving asthma with anti-inflammatory and anti-allergic effects.

## Supplementary Information

Supplementary Material 1.

## Data Availability

The datasets used and analyzed during the current study are available from the corresponding author on reasonable request.

## Abbreviations

CON: Control
OVA: Ovalbumim
GEB: Gastrodia elata Blume
NK: Natural killer
HPLC-DAD: High-performance liquid chromatography-diode array detector
Alum: Aluminum hydroxide
ELISA: Enzyme-linked immunosorbent assay
IHC: Immunohistochemistry
HRP: Horseradish peroxidase
BW: Body weight
SST: Serum seperation tube
BALF: Bronchoalveolar lavage fluid
PBS: Phosphate-buffered saline
i.p.: Intraperitoneal injection
i.n.: Intranasal instillation
Th2: T helper 2
IL-4: Interleukin 4
IL-5: Interleukin 5
IL-13: Interleukin 13
H&E: Hematoxylin and eosin
PAS: Periodic acid-Schiff
DAB: 3,3'-Diaminobenzidine tetrahydrochloride
SD: Standard deviation
