# Supplementary material for: Anti-allergic and anti-inflammatory effects of Gastrodia elata Blume extract in ovalbumin-induced asthma rat model
Source: Lab Anim Res. 2025 Sep 16;41:21. doi: 10.1186/s42826-025-00252-8 (PMC12439423; doi:10.1186/s42826-025-00252-8)

Group (n=8)	Intraperlitoneal injection (I.P.)	Intranasal instillation (I.N.)	Per oral (P.O.)
CON	Alum 10 mg in saline (1 mL)	Saline (100 µL)	DW (10 mL/kg/day)
OVA	Alum 10 mg + OVA 200 µg in saline (1 mL)	1% OVA in saline (100 µL)	
GEB			GEB (7 g/kg/day) in DW

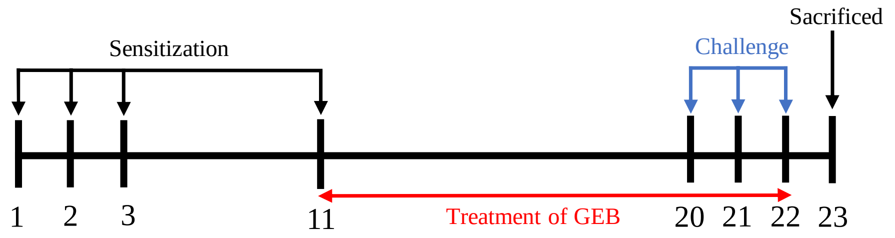

Supplement: Supplementary file 1 — Supplementary Material 1. [file 42826_2025_252_MOESM1_ESM.pdf]
